# Experiences of primary care physicians and staff following lean workflow redesign

**DOI:** 10.1186/s12913-018-3062-5

**Published:** 2018-04-10

**Authors:** Dorothy Y. Hung, Michael I. Harrison, Quan Truong, Xue Du

**Affiliations:** 1 Palo Alto Medical Foundation Research Institute, 2350 W. El Camino Real #447, Mountain View, CA 94040 USA; 2 Agency for Healthcare Research and Quality, Center for Delivery, Organization, and Markets, 5600 Fishers Lane, Mail Stop 7W25B, Rockville, MD 20857 USA; 3 Palo Alto Medical Foundation Research Institute, 2350 W. El Camino Real #4012, Mountain View, CA 94040 USA; 4 Palo Alto Medical Foundation Research Institute, 2350 W. El Camino Real #4014, Mountain View, CA 94040 USA

**Keywords:** Primary care redesign, Patient care team, Work environment, Professional burnout, Organizational innovation

## Abstract

**Background:**

In response to growing pressures on primary care, leaders have introduced a wide range of workforce and practice innovations, including team redesigns that delegate some physician tasks to nonphysicians. One important question is how such innovations affect care team members, particularly in view of growing dissatisfaction and burnout among healthcare professionals. We examine the work experiences of primary care physicians and staff after implementing Lean-based workflow redesigns. This included co-locating physician and medical assistant dyads, delegating significant responsibilities to nonphysician staff, and mandating greater coordination and communication among all care team members.

**Methods:**

The redesigns were implemented and scaled in three phases across 46 primary care departments in a large ambulatory care delivery system. We fielded 1164 baseline and 1333 follow-up surveys to physicians and other nonphysician staff (average 73% response rate) to assess workforce engagement (e.g., job satisfaction, motivation), perceptions of the work environment, and job-related burnout. We conducted multivariate regressions to detect changes in experiences after the redesign, adjusting for respondent characteristics and clustering of within-clinic responses.

**Results:**

We found that both physicians and nonphysician staff reported higher levels of engagement and teamwork after implementing redesigns. However, they also experienced higher levels of burnout and perceptions of the workplace as stressful. Trends were the same for both occupational groups, but the increased reports of stress were greater among physicians. Additionally, members of all clinics, except for the pilot site that developed the new workflows, reported higher burnout, while perceptions of workplace stress increased in all clinics after the redesign.

**Conclusions:**

Our findings partially align with expectations of work redesign as a route to improving physician and staff experiences in delivering care. Although teamwork and engagement increased, the redesigns in our study were not enough to moderate long-standing challenges facing primary care. Yet higher levels of empowerment and engagement, as observed in the pilot clinic, may be particularly effective in facilitating improvements while combating fatigue. To help practices cope with increasing burdens, interventions must directly benefit healthcare professionals without overtaxing an already overstretched workforce.

**Electronic supplementary material:**

The online version of this article (10.1186/s12913-018-3062-5) contains supplementary material, which is available to authorized users.

## Background

Primary care faces growing pressure to deliver higher quality care at lower cost, a trend accelerated in the United States by recent expansion of insurance coverage and embodied in the growth of value-based payment [1, 2]. Demand for primary care change in the U.S and other industrial countries reflects budgetary constraints, population ageing, growing chronic illness, and reliance on primary care to reduce use of more costly services [3]. In response, leaders have advocated and introduced a wide range of primary care workforce innovations, including some that redesign the entire practice. These redesigns create teams in which more time is allocated for managing chronic and preventive care, and where allied health practitioners relieve physicians of routine tasks [4–6].

One important question is how such care redesigns affect physicians and other members of primary care teams. This issue is particularly important in view of growing evidence on physician dissatisfaction and burnout, and concern over shortages of primary care physicians and nurses [3, 7]. Some analysts anticipate that if physicians’ tasks are shared by other team members, physicians will experience greater work satisfaction and less burnout [1, 8]. Observations of high-functioning primary care practices support this expectation [9], as do some studies on implementing the patient-centered medical home (PCMH) [10, 11]. There is evidence that a diverse range of changes in primary care, including work redesigns, can reduce burnout and enhance satisfaction [12–14], but limited information on how these changes affect particular types of physicians and also nonphysician staff.

Further research is clearly needed to illuminate how physicians and other staff experience care redesign and how diverse settings impact these experiences. To help address these questions, we report here on a study of a primary care redesign that grew out of an application of Lean management principles, which were derived from the manufacturing industry. Lean’s basic premise is to improve efficiency by removing waste and streamlining flow [15, 16]. This is accomplished through the mapping of start-to-end processes, called “value stream mapping,” to identify non-value-added activities. More broadly Lean aims to empower and engage the workforce in continuous quality improvement [16–18]. This type of approach has been shown to improve healthcare delivery [16, 19–24] and a wide range of operational metrics such as efficiency, productivity, and satisfaction among patients, providers and staff, without detriments to clinical quality [25]. But less is known about Lean’s influence on teamwork, staff engagement, and participation in quality improvement, all of which are also targeted by Lean management principles.

To contribute to the broader study of care redesign and add to knowledge of Lean in primary care, we examine physician and staff experiences after implementing a series of workflow changes. Based on the goals of Lean methodology and previous findings from inpatient settings [24, 26], we expect work redesigns to increase staff empowerment, engagement, and satisfaction among primary care physicians and other care team members. We also expect that Lean redesigns can reduce burnout and workplace stress among staff. However, we recognize some analysts expect that Lean applications could lead to a speed-up of care and reductions in autonomy [27, 28], which may ultimately result in higher burnout and work dissatisfaction. These issues are considered within our broader study of work experiences among primary care physicians and nonphysician staff.

## Methods

### Study setting and data source

This study was conducted in a not-for-profit, ambulatory care system in the U.S serving over one million patients across six counties. In efforts to improve performance, the delivery system implemented Lean-based redesigns beginning in the area of primary care. The redesigns were implemented in all primary care departments using the same sequence of activities: (1) “5S” standardization of medical equipment, supplies, and health education materials in patient exam rooms; (2) call management and redesign of call center functions; (3) co-location of existing care teams composed of a physician and a medical assistant (MA); and (4) redesign of care team roles and workflows, including daily huddles between physician-MA dyadic care teams, agenda setting by MAs at the start of patient visits, and designation of all MAs as care team “Flow Managers” responsible for managing or triaging incoming patient care items (e.g., test results, referrals, patient messages). Over an implementation period of 2 years, daily workflows for all physicians and staff were redesigned in 46 primary care departments located within 17 geographically distinct clinics across the system. Each clinic location housed 1 to 3 primary care departments, i.e., Family Medicine, Internal Medicine, and/or Pediatrics. The implementation occurred in three phases, starting with primary care departments in one pilot clinic (phase 1), followed by three “beta” test clinics (phase 2), and completed in all 13 remaining clinics (phase 3). During the pilot phase, physicians and other staff were deeply engaged in the design of new work roles and workflows. These changes were implemented in the beta clinics with some further modifications, and then spread to the remaining clinics by organizational leaders with the support of external Lean consultants.

Prior to implementation, a team of independent researchers embedded within the delivery system fielded a baseline survey to 1333 primary care physicians and non-physician staff (e.g., MAs, licensed vocational nurses, patient service representatives) across the system. The organization’s Institutional Review Board (IRB) approved all data collection activities, which included voluntary completion of work experience surveys. The average response rate for the baseline survey was 73%, with a range of 63%–86% by clinic location. A follow-up survey was administered in the same clinics to 1164 physicians and staff upon completion of redesigns. Based on the sequential phases of implementation, this follow-up survey was administered 2.5 to 3 years after completion of redesigns in the pilot clinic, 1.5 to 2 years after completing redesigns in phase 2 clinics, and 8 to 5 months after redesigns were completed in phase 3 clinics. The follow-up had an average response rate of 74%, ranging from 67%–77% by clinic location. Both surveys assessed current experiences of work, including levels of employee engagement and job-related burnout; and perceptions of work environments such as degree of teamwork, participation in decision making, and levels of stress in the workplace.

### Work experience measures

#### Physician and staff engagement

We assessed employee engagement among physicians and staff using an adapted version of a work experience survey [29]. As this instrument lacked specification of a priori domains, we conducted exploratory factor analysis with varimax rotation that yielded three separate factors (3 items per domain, eigenvalues > 1) with Cronbach’s alpha coefficients of 0.89, 0.84 and 0.81, respectively. We labeled these factors as: ***(1) Personal motivation***, perceptions among staff that work contributions are valued and that professional development is encouraged (e.g., “My ideas and suggestions for improvement are valued by my department,” “My manager provides me with sufficient opportunities to improve myself”); ***(2) Work satisfaction****,* degree to which individuals are satisfied in the workplace (e.g., “Overall, I think this is a great place to work”); and ***(3) Ownership***, degree to which individuals contribute to and understand how their efforts affect the organization’s goals (e.g., “I am willing to put in a great deal of effort to help my department succeed,” “I understand how my daily work contributes to my department’s mission”).

#### Work environment

We assessed perceptions of the work environment using a validated instrument developed to measure organizational attributes in primary care practices [30]. This instrument has three subscales of 3–5 items each, all demonstrating high internal consistency and reliability with Cronbach’s alpha coefficients of 0.76, 0.74, and 0.71. The following attributes were assessed in each department: ***(1) Teamwork***, the extent to which members from different organizational levels and job functions work together as a team (e.g., “Staff and clinicians in this department operate as a real team”); ***(2) Participation in Decision Making*** or collective problem solving (e.g., “All staff members participate in important decisions about clinical operations”); and ***(3) Workplace Stress***, reflecting negative feelings about workload and operating conditions (e.g., “This department is experienced as ‘stressful’,” “It’s hard to make any changes in this department because we’re so busy seeing patients”).

All measures of engagement and work environment used 5-point Likert scales ranging from 1 = “strongly disagree” to 5 = “strongly agree.” Scores for each domain were averaged for each respondent.

#### Job-related burnout

The well-validated Maslach Burnout Inventory (MBI—Human Services Version) [31] was used to assess levels of burnout and how health professionals view their daily work activities. We used the MBI to measure three domains each with 5–7 items and Cronbach’s alpha coefficients of 0.91, 0.70, and 0.76, respectively: ***(1) Emotional exhaustion*** or fatigue from delivering patient care (e.g., “I feel emotionally drained from my work”); ***(2) Depersonalization***, a hardening of the attitudes of care providers toward patients (e.g., “I feel I treat some patients as if they were impersonal objects”); and ***(3) Personal accomplishment***, a positive self-assessment of care provision (e.g., “I feel I’m positively influencing other people’s lives through my work”). All statements were assessed on 7-point scales ranging from 1 = “never” to 7 = “every day,” and averaged for each respondent.

#### Respondent characteristics

Demographic and other respondent characteristics included: gender, age, ethnicity, race, education, departmental tenure, professional role, and clinic phase for implementing Lean redesigns. The variables were categorized with reference groups: female, over 60, non-Hispanic, white, more than 4-year college degree, less than 1-year tenure, physician, and pilot clinic (phase 1 implementation). Based on two sample independent t-tests, the two statistically significant differences in respondent characteristics between baseline and follow-up cohorts were respondent race and departmental tenure.

### Statistical analysis

We first conducted multivariate regression analyses to detect changes in work experiences between baseline and follow-up surveys, adjusting for respondent characteristics and response clustering within departments in each clinic-location. To assess need for hierarchical modeling, intercept-only models were first used to calculate intra-class correlations (ICC) for each outcome variable. All ICC values were above 0.10 suggesting the appropriateness of the hierarchical approach [32]. All regression analyses thus leveraged random intercept models to account for the hierarchical (i.e., nested) structure of individuals located within departments. The main covariate of interest in each regression model was a binary variable indicating follow-up, as compared with baseline, experiences as reported on physician and staff surveys. Robust standard errors were estimated in all models to avoid violating the assumption of independence due to correlated observations within departments. All models were fitted excluding null values or outliers identified in diagnostic plots of residuals. Statistical analyses were conducted using R version 2.11.1 and SAS Enterprise Guide 5.1.

## Results

Table 1 presents univariate results for both baseline and follow-up survey respondents. There were no structural differences between cohorts except for tenure in the department and distribution of respondent race/ethnicity.

**Table 1:** Characteristics of Survey Respondents

	Baseline (*N* = 970)		Follow-up (*N* = 860)	
	*N*	%	*N*	%
Gender				
Female	770	79%	599	70%
Male	169	17%	122	14%
Age				
Under 30	145	15%	113	13%
30–39	313	32%	212	25%
40–49	255	26%	213	25%
50–59	181	19%	143	17%
60 or over	49	5%	43	5%
Ethnicity				
Non-Hispanic	689	71%	400	47%
Hispanic	205	21%	122	14%
Race*				
Black or African American	22	2%	7	1%
American Indian or Alaska Native	3	0.3%	5	1%
Native Hawaiian or Pacific Islander	18	2%	20	3%
Asian	221	23%	129	16%
White (European, Middle Eastern)	437	45%	284	34%
Other	207	21%	40	5%
Education				
Some high school, High school graduate/GED	71	7%	53	6%
Some college or 2-year degree	394	41%	274	32%
4-year college degree	99	10%	74	9%
More than 4-year college degree	392	40%	330	38%
Tenure in Department*				
Less than 1 year	105	11%	78	9%
1–2 years	99	10%	113	13%
2–5 years	278	29%	140	16%
More than 5 years	478	49%	414	48%
Specialty				
Family Medicine	290	30%	328	38%
Internal Medicine	261	27%	280	33%
Pediatrics	235	24%	252	29%
Professional Role				
Physician	350	36%	330	38%
Non-Physician	518	53%	521	61%
Phase of Lean Implementation				
Pilot (phase 1)	133	14%	99	12%
Beta (phase 2)	328	34%	270	31%
Gamma (phase 3)	509	52%	491	57%

Note: Cells may not add to 100% due to missing data

*p < 0.05

**Fig. 1:**
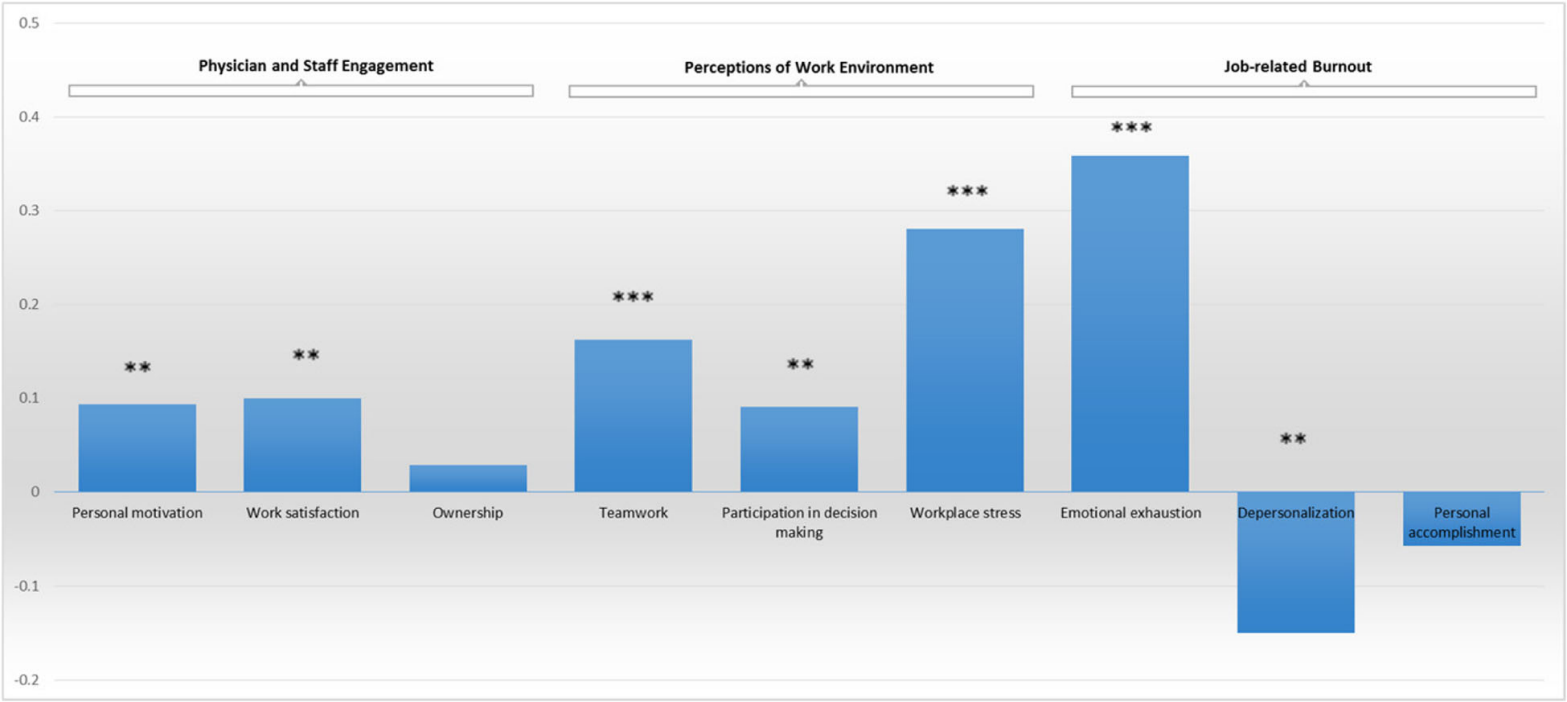
Changes in Physician and Staff Experiences After Lean Workflow Redesigns. Reference group: Baseline survey. Note: All models are adjusted for respondent characteristics, including gender, age, race/ ethnicity, education, department tenure, and clinic phase of implementation. **p < 0.05, ***p < 0.01

As shown in Fig. 1, there were improvements in reported measures of engagement, teamwork, and participation in decision-making among physicians and staff after implementing Lean changes. However, reports of workplace stress (a measure of work environment) and emotional exhaustion (a measure of burnout) also dramatically increased. Since there were some differences between the experiences of physicians and nonphysician staff, we present these results separately in Tables 2 and 3.

**Table 2:** Changes in Physician Experiences After Workflow Redesigns (*N* = 680)

Experience Domains	Parameter Estimate^a^	Standard Deviation
Physician Engagement		
Personal motivation	0.065	0.076
Work satisfaction	0.122*	0.089
Ownership	0.091*	0.064
Perceptions of Work Environment		
Teamwork	0.200***	0.060
Participation in decision making	0.100*	0.077
Workplace stress	0.406***	0.068
Job-related Burnout		
Emotional exhaustion	0.390***	0.135
Depersonalization	−0.018	0.112
Personal accomplishment	−0.154**	0.069

Note: All models are adjusted for respondent characteristics, including gender, age, race/ ethnicity, education, department tenure, and clinic phase of implementation

^a^Reference group: Baseline survey

*p < 0.10, **p < 0.05, ***p < 0.01

**Table 3:** Changes in Nonphysician Staff Experiences After Workflow Redesigns (*N* = 1039)

Experience Domains	Parameter Estimate^a^	Standard Deviation
Staff Engagement		
Personal motivation	0.115**	0.070
Work satisfaction	0.076	0.066
Ownership	−0.005	0.052
Perceptions of Work Environment		
Teamwork	0.146**	0.059
Participation in decision making	0.096*	0.069
Workplace stress	0.177***	0.067
Job-related Burnout		
Emotional exhaustion	0.365**	0.120
Depersonalization	−0.244***	0.077
Personal accomplishment	0.034	0.097

Note: All models are adjusted for respondent characteristics, including gender, age, race/ ethnicity, education, department tenure, and clinic phase of implementation

^a^Reference group: Baseline survey

*p < 0.10, **p < 0.05, ***p < 0.01

Physicians reported increases in two of the three engagement measures: work satisfaction and ownership. There were no improvements at follow-up for nonphysician staff in these dimensions, but there was growth in personal motivation, another measure of engagement. Positive trends in perceptions of the work environment, including teamwork and participation in decisions to improve care, were the same for both groups. Nonphysician staff reported greater work stress, as did physicians, but the increase from baseline was much greater among physicians. Both occupational groupings also experienced higher levels of emotional exhaustion after the redesigns, but only physicians reported lower feelings of personal accomplishment. In contrast, after the redesigns, staff other than physicians reported less depersonalization of relations to patients than had originally been the case.

All work experience results shown in Tables 2 and 3 were adjusted for respondent characteristics. Briefly, respondents of younger age (less than 60) and with longer tenure in the department (over 2 years in most cases) reported less engagement, teamwork and motivation, along with more stress and burnout compared to the reference groups (i.e., those over 60 and those with less than 1 year tenure). Non-physicians with less education reported less emotional exhaustion and workplace stress, along with more personal accomplishment and work satisfaction compared to those with more than a four-year degree. Race was significant with African American physicians reporting less work satisfaction, while Asian and Native Hawaiian/Pacific Islander physicians and non-physician staff reported more motivation and less burnout, than white physicians. Among physicians, males reported more personal motivation, teamwork, and work satisfaction than females. These results can be found in the Additional files 1 and 2.

To further explore the growth in workplace stress and burnout observed in Tables 2 and 3, we examined relationships between changes in work experiences and specific phase in which clinics implemented the redesigns. Implementation phase provided a rough indicator of how long respondents were exposed to the new workflows. There were no significant differences in stress or burnout levels between either phase 2 or phase 3 clinics and the pilot site. Hence, changes in work stress and burnout do not appear to reflect length of exposure to the redesigns. To explore these changes further, we conducted three separate sets of regression analyses for each distinct cohort of clinics according to implementation phase. As shown in Table 4, in all three phases perceptions of workplace stress were higher after the redesign than beforehand. Members of all clinics except for the pilot also reported higher burnout at follow-up compared to baseline. In contrast, members of the pilot clinic reported a higher sense of personal accomplishment from work and no changes on the other two measures of burnout after implementing Lean redesigns.

**Table 4:** Changes in Workplace Stress and Burnout After Redesigns in Each Phase (Separate Analyses, by Implementation Phase)

	Pilot (Phase 1) (*N* = 232)		Betas (Phase 2) (*N* = 558)		All Other Clinics (Phase 3) (*N* = 1000)	
Experience Domains	Parameter Estimate^a^	(SD)	Parameter Estimate^a^	(SD)	Parameter Estimate^a^	(SD)
Perceptions of Work Environment						
Workplace stress	0.419***	(0.144)	0.332***	(0.090)	0.231***	(0.063)
Job-related Burnout						
Emotional exhaustion	−0.016	(0.248)	0.476***	(0.164)	0.383***	(0.120)
Depersonalization	−0.016	(0.207)	−0.230*	(0.118)	−0.117	(0.087)
Personal accomplishment	0.417*	(0.222)	−0.308***	(0.095)	−0.001	(0.086)

Note: All models adjusted for respondent characteristics, including gender, age, race/ethnicity, education, department tenure, and clinic implementation phase

SD, Standard Deviation

^a^Reference group: Baseline survey

*p < 0.05, **p < 0.01, ***p < 0.001

## Discussion

We examined differences in work experiences reported by physicians and staff after a system-wide redesign in primary care clinics. The redesign, which was based on Lean workflow principles, co-located MA-physician dyads, delegated significant responsibilities to MAs, and mandated greater coordination and communication among care team members. After implementation of Lean redesigns, physicians and staff reported higher levels of teamwork and more participation in decisions to improve care. Physicians also reported higher levels of engagement, including increased work satisfaction and ownership, while nonphysician staff reported becoming more personally motivated at work. However, both physicians and other staff experienced increased burnout at follow-up, and reported higher workplace stress than at baseline.

These mixed findings partially align with the expectations of researchers who view work redesign as a route to improving the experience of primary care providers. In our study, teamwork, participation, and work engagement rose among physicians and staff, as might have been anticipated from other studies of primary care redesign and from research on Lean in hospital settings [9, 33–36]. These findings may have reflected specific benefits of co-locating each dyadic care team in a shared workspace. Besides improving workflow efficiency, this feature of the redesign served to enhance communication and partnership between physicians and MAs. Additionally, formal re-designation of MAs as Lean “Flow Managers” may have yet furthered a sense of teamwork and participation in decisions; in this new role, MAs became responsible for managing daily team workflows and incoming patient care items.

These findings are aligned with past observations of primary care redesigns that found benefits in proximity of workstations, and that “shared-care models” improved satisfaction among physicians and staff [9, 33, 34]. However, although these activities likely increased experiences of teamwork and participation in the work process, these improvements did not significantly enhance nonphysicians’ sense of ownership or overall work satisfaction as observed among physicians. Similarly, other researchers who studied a number of primary care practices found the transition to team-based care “does not come naturally” and, in fact, may be particularly challenging among certain health professionals [37]. It may be that other aspects of physician–staff interactions remain unaffected by redesigns and continue to reinforce certain experiences among nonphysicians with the care delivery process.

Unlike our research, prior studies have not typically explored whether new team functions may lead to unintended consequences like burnout. We found that despite their positive effects, Lean redesigns did not relieve work burdens reported by both physicians and non-physician staff. On the contrary, after the redesigns both groups reported significantly higher levels of workplace stress and some forms of burnout than they did beforehand. Lean efficiencies have been found in other care settings to “give the gift of time” [38], but this was not apparent in our study of primary care clinics. Although reported work experiences may reflect demands imposed by this organization as it sought to increase efficiency, it is also entirely possible that they reflect secular trends such as the growing burden on primary care providers. This was likely aggravated during our study period as pressure grew to manage complex patients and as insurance coverage expanded under the Affordable Care Act, which passed and was implemented contemporaneously with the Lean initiative at this study organization. Hence, our study may serve to highlight unique differences between primary care and other healthcare settings that have adopted Lean, potentially reflecting distinctive challenges facing primary care. Consistent with this, other studies indicate that primary care may be especially vulnerable to increasing workloads and decreased satisfaction after initiation of new designs [39].

Our study may also reflect features of primary care change that make it difficult, but not impossible, to obtain benefits of teamwork and task delegation as reported in some studies. One possibility is suggested by the contrast between our findings and those of Linzer and colleagues [13]. In their research, physicians whose practices were randomly selected for an intervention group were encouraged to choose among a variety of possible workflow designs targeting known predictors of burnout, and then to develop quality improvement teams to implement them based on their own practice data. In our study, members of the pilot team also were deeply engaged in developing new workflows; however, members of the phase 2 beta sites and all remaining phase 3 sites had less discretion, since they were charged mainly with either adopting or adapting designs to fit their local environments. The difference in empowerment between the majority of clinics in our study and those in the experiment by Linzer and colleagues [13] may help explain the divergence in burnout outcomes. In like manner, greater empowerment of the pilot clinic members in our study may explain why they did not experience more burnout after implementing changes. It may be that staff engagement is particularly effective in facilitating improvements while also combating fatigue.

Finally, some limitations of our study include the lack of a comparison group as the organization implemented Lean redesigns in all clinic sites. Thus, we cannot determine with certainty whether observed changes were due to secular trends or to the Lean redesign itself. Nor was our study based on a panel design, though we do provide a comparison of respondent characteristics from the baseline and follow-up surveys. Based on these results and adjusted regression models, it is unlikely that self-selection or workforce composition skewed the differences observed in pre- and post-Lean work experiences. Finally, closed-ended questions on the MBI were not detailed enough for us to identify specific causes of reported burnout. Despite this limitation, we attempted to distinguish between Lean-caused fatigue versus secular trends in primary care by exploring patterns between pilot clinic, phase 2 and phase 3 implementation sites.

## Conclusions

Leveraging operational activities and survey data in a large ambulatory care delivery system, we sought to understand work experiences of primary care physicians and staff after implementing workflow redesigns that held promise for fostering teamwork, communication, and workflow efficiency for all clinic staff. The redesigns were also intended to reduce workload and burnout among physicians by delegating some tasks to other staff members. Following implementation of changes, we indeed observed higher levels of teamwork, participation and engagement, but we also found higher levels of workplace stress and burnout. Taken together, our results provide a complex picture of the impact of Lean on primary care physicians and staff. Despite the largely favorable responses among survey respondents, our study suggests that Lean-based redesigns have not yet reduced growing challenges in primary care, where a shortage of physicians and growing patient population create dual burdens on care delivery. To help primary care practices cope with existing challenges, changes must create efficiencies that directly benefit care providers without overtaxing an already overstretched workforce.

## Additional files

Additional file 1: Respondent Characteristics When Assessing Changes in Physician Experiences After Workflow Redesigns. This file details all the respondent characteristics that were adjusted for in the analysis of physician work experiences shown in Table 2. (DOCX 601 kb)

Additional file 2: Respondent Characteristics When Assessing Changes in Non-physician Staff Experiences After Workflow Redesigns. This file details all the respondent characteristics that were adjusted for in the analysis of non-physician staff work experiences shown in Table 3. (DOCX 562 kb)

## Abbreviations

HMO: Health maintenance organization
ICC: Intra-class correlations
MA: Medical assistant
MBI: Maslach Burnout Inventory
PCMH: Patient-centered medical home
