# Supplementary material for: Experiences of primary care physicians and staff following lean workflow redesign
Source: BMC Health Serv Res. 2018 Apr 10;18:274. doi: 10.1186/s12913-018-3062-5 (PMC5894127; doi:10.1186/s12913-018-3062-5)
Supplement: Supplementary file 1 — Respondent Characteristics When Assessing Changes in Physician Experiences After Workflow Redesigns. This file details all the respondent characteristics that were adjusted for in the analysis of physician work experiences shown in Table 2. (DOCX 601 kb) [file 12913_2018_3062_MOESM1_ESM.docx]

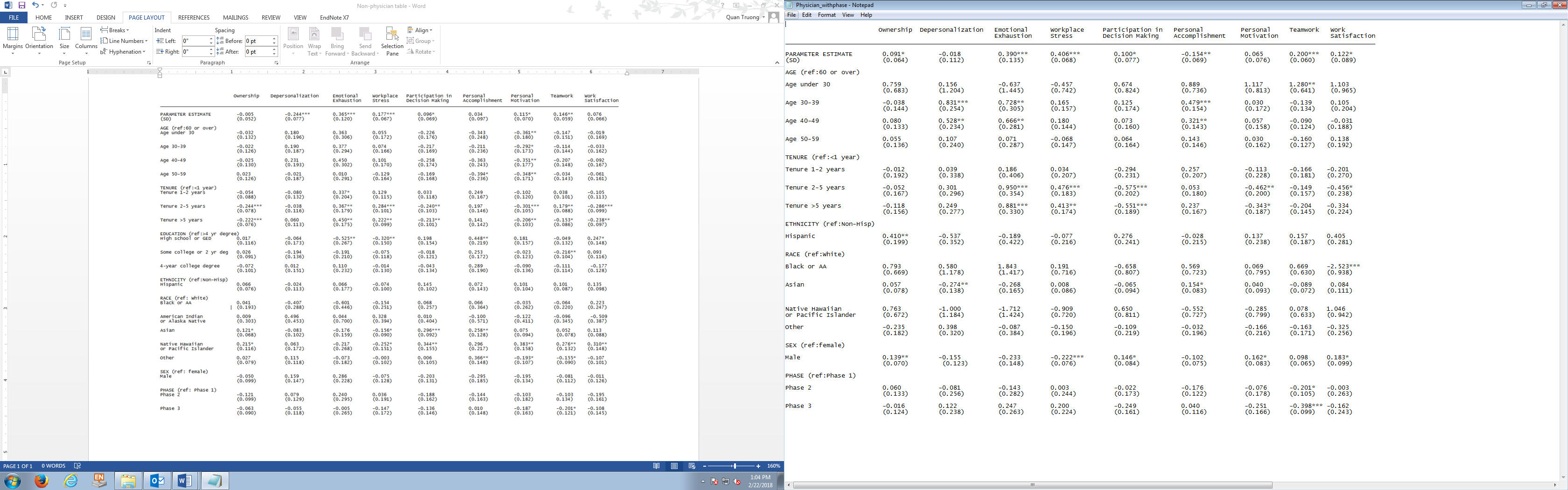
**Additional file 1 Respondent Characteristics When Assessing Changes in Physician Experiences After Workflow Redesigns**
