# Supplementary material for: Experiences of primary care physicians and staff following lean workflow redesign
Source: BMC Health Serv Res. 2018 Apr 10;18:274. doi: 10.1186/s12913-018-3062-5 (PMC5894127; doi:10.1186/s12913-018-3062-5)

**Additional file 2 Respondent Characteristics When Assessing Changes in Non-physician Staff Experiences After Workflow Redesigns**


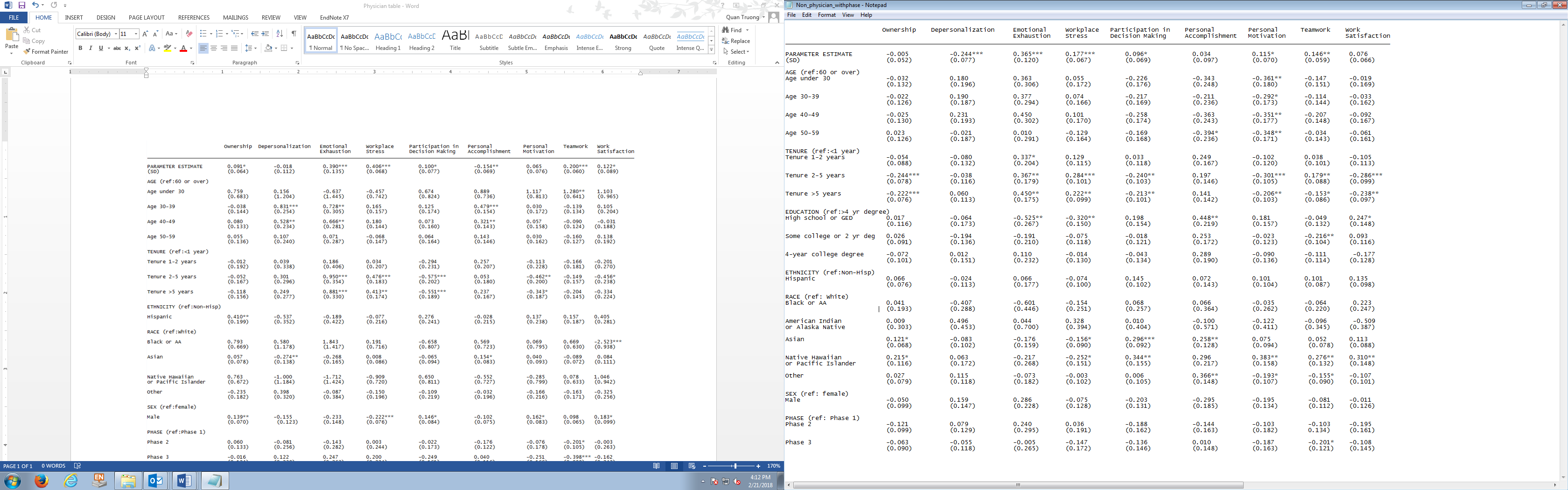

Supplement: Supplementary file 2 — Respondent Characteristics When Assessing Changes in Non-physician Staff Experiences After Workflow Redesigns. This file details all the respondent characteristics that were adjusted for in the analysis of non-physician staff work experiences shown in Table 3. (DOCX 562 kb) [file 12913_2018_3062_MOESM2_ESM.docx]
